# Effects of Hormone Replacement Therapy on Insulin Resistance in Postmenopausal Diabetic Women

**DOI:** 10.3889/oamjms.2016.024

**Published:** 2016-02-01

**Authors:** Iskra Bitoska, Branka Krstevska, Tatjana Milenkovic, Slavica Subeska-Stratrova, Goran Petrovski, Sasha Jovanovska Mishevska, Irfan Ahmeti, Biljana Todorova

**Affiliations:** *University Clinic of Endocrinology, Diabetology and Metabolic Disorders, Faculty of Medicine, Ss Cyril and Methodius University of Skopje, Skopje, Republic of Macedonia*

**Keywords:** Hormone replacement therapy, Menopause, Diabetes mellitus, Insulin resistance, HOMA - IR

## Abstract

**BACKGROUND:**

Insulin resistance (IR) is closely associated with diabetes mellitus. On the other hand, increased visceral fat in menopause is also associated with IR, which makes postmenopausal diabetic women in a big risk for cardiovascular diseases. There are conflicting reports about the effects on hormone replacement therapy (HRT) on IR.

**AIM:**

The aim of the study was to investigate the effects of HRT on IR.

**METHODS:**

A total of 40 postmenopausal women with type 2 diabetes were enrolled and followed for 12 months. Half of them were assigned to take HRT, while the other half made the control group. Fasting plasma glucose (FPG) and insulinemia were measured in both groups at baseline and after 12 months. IR was represented by Homeostatic model assessment for IR (HOMA-IR).

**RESULTS:**

HRT was associated with significant decrease in HOMA-IR, FPG and insulinemia in the examined group. There was no significant reduction in FPG and no significant increase in insulinemia levels and HOMA-IR values in control group after 12 months.

**CONCLUSION:**

HRT was associated with statistically signifficant increase of insulin sensitivity. Larger clinical trials will be necessary to understand whether HRT may improve insulin resistance and glucose homeostasis in women with diabetes, especially when given shortly after entering menopause.

## Introduction

Postmenopausal estrogen therapy and estrogen plus progesterone hormone replacement therapy (HRT) alleviate symptoms of menopause and attenuate bone loss [1]. Moreover, several observational studies suggest that use of estrogen replacement therapy decreases the risk of coronary heart disease [2-4] and lowers overall mortality rates [5, 6].

The menopause transition, as well as the early postmenopausal period, is associated with an increase in total and central obesity [7-9]. Increased visceral fat is associated with insulin resistance [10], and this preferential storage of abdominal fat may contribute to cardiovascular disease and diabetes in postmenopausal women. Estrogen and HRT may improve fat distribution in postmenopausal women by preventing the increase in central body fat [7, 11-13]. However, the evidence concerning the effects of HRT on glucose homeostasis is controversial. Estrogen replacement therapy has been reported to have no effect on insulin sensitivity in postmenopausal women [14-16] and to improve carbohydrate metabolism in individuals with type 2 diabetes [18, 19]. In contrast, nondiabetic women taking estrogen alone were more insulin-resistant than women not on HRT or women taking estrogen and HRT [19]. Differences in the study population, type and route of administration of hormone therapy, and method of measuring insulin sensitivity may explain the disparate results of estrogen replacement on glucose metabolism.

Individuals with insulin resistance require increasing levels of insulin to maintain normal glucose levels and are likely to progress to type 2 diabetes mellitus [20]. Insulin resistance is often measured by using the homeostasis model assessment–insulin resistance (HOMA-IR) equation, which is a function of the product of fasting insulin and glucose levels [21]. Fasting insulin levels are the major indicator of insulin resistance and are increased by obesity and decreased with higher degree of physical activity [22]. Despite the potential advantages of measures of insulin resistance, the prognostic value of such measurements has not been extensively evaluated. Estimation of insulin resistance using either levels of insulin, HOMA-IR, glucose, or the ratio of triglycerides and high-density lipoprotein-cholesterol (TG/HDL-C) could potentially improve on cardiovascular risk stratification [23].

Insulin resistance and hyperinsulinemia are clinically important since the effects of insulin have been shown in the formation of atherosclerotic plaques, [24] and, hence, increased risk of hypertension and atherosclerosis has been attributed to insulin resistance in postmenopausal women. In these women, fasting glucose and insulin levels decrease with HRT [25]. It has been postulated that insulin resistance could decrease with HRT use [26]. Different studies resulted in different conclusions; estrogen and progesteron were shown to decrease insulin sensitivity in one study [27], but were reported not to affect insulin sensitivity in others [27, 28].

For this reason, we aimed to study the effects of HRT on IR by the HOMA - IR

## Patients and Methods

We prospectively studied 40 women in natural menopause with type 2 diabetes. Diabetes was diagnosed using the criteria of the World Health Organization, at least 2 years before entering the study. In order to maintain their glucose levels in an acceptable range, women with type 2 diabetes were on dietary management alone (two patients) or taking oral anti-diabetic drugs that consisted of metformin and sulfonylureas (38 patients). Each diabetic patient received a diabetic diet of 1300 ckal/day. The women were instructed not to change their diet. None of them were taking insulin. None of the women were taking anti-lipidaemic, corticosteroid or anti-convulsant therapy. The anti-diabetic medications were left unchanged during the study.

Menopause was confirmed by the absence of menstruation for at least 12 months and by high serum levels of FSH (> 30 mIU/ml) and low serum levels of estradiol (E_2_) (< 20 pg/ml). The subjects had not received HRT previously. Gynaecological examination and mammogram were normal in all subjects.

Half of the subjects (20 women) were assigned to take HRT (DM – HRT) group. The other half (20 women) made the control group, not taking HRT (DM – non HRT group). The randomisation of the subjects has been done upon base of willingness and motivation to cooperate. Subjects in the DM - HRT group had been taking oral HRT consisting of 17β - estradiol (E2) 1 mg and DRSP (drospirenone) 2 mg - Angeliq®, Schering AG, Germany, 1 tablet daily for 12 months. Subjects in DM – non HRT group were followed the same way as examined group. Exclusion criteria in both groups included *1*) hypertension (systolic blood pressure ≥ 140 or diastolic blood pressure ≥ 90 mmHg); *2*) anemia; 3) various degrees of renal insufficiency; 4) evidence of significant liver disease; and 5) hysterectomy or a history of recent surgery and who demonstrated significant chronic alcohol intake, were also excluded. All patients in this study gave written informed consent.

This work was approved by the local medical ethics committee and all participants gave informed consent before the onset of study.

All of the participants enrolled in our study have been contacted by telephone in three months + interval in order to discover any adverse effect of HRT. All metabolic and physical examinations were performed at the onset of the study and then again after 12 months of receiving HRT. Blood samples were taken after a 12 h fast. HbA_1_C was determined on an Cobas c 111 analyzer using commercial kits supplied from Roche Diagnostic Gmbh (Switzerland), and glucose levels were determined in an Beckman Analyzer 2 automated analyser by using commercial kits supplied from Analox Instruments Ltd, London (UK). Insulinemia was determined in an Elecsys 2010 analyzer using commercial kits from Roche Diagnostics Gmbh (Switzerland).

### Assessment of IR

In primary analysis, we evaluated degrees of IR by homeostatic model assessment for IR (HOMA-IR) using the formulas HOMA-IR = glucose × insulin/22.5. A higher HOMA-IR value indicates greater IR.

### Statistical analysis

Statistical analysis was carried out with descriptive statistics, t - test for related samples and t - test for independent samples. The data are expressed as means ± SEM. Statistical significance was set at *P* < 0.05. Data were analysed using Statistica, version 10.0 (StatSoft).

## Results

All of the women who were enrolled into the investigation completed the study. The mean age of the subjects was 49.3 ± 0.34 and 48.5 ± 3.1 years, and their mean body mass index (BMI) was 27.27 ± 3.32 and 28.3 ± 2.4 kg/m^2^ in the DM-HRT and DM non-HRT groups respectively. High school educated was 58.3 % and 54.2 %. The baseline characteristics of the subjects are given in Table 1. Two subjects in the DM-HRT group and one subject in the DM non-HRT group complained about abnormal vaginal bleeding such as metrorrhagia and four patients reported breast tenderness in the DM-HRT group. Other adverse effects were not seen

**Table 1:** Baseline characteristics of postmenopausal women by HRT status

	Women on HRT (*n* = 20)	Women not on HRT (*n* = 20)	P
Age (years)	49 ± 3.34	48.5 ± 3.1	N/S
BMI (kg/m^2^)	27 ± 3.32	28.3 ± 2.4	N/S
Fasting plasma glucose (mmol/l)	7.8 ± 0.86	8.0 ± 0.9	N/S
HbA1C (%)	7.6 ± 0.54	7.9 ± 0.5	N/S
Insulinemia(µU/ml)	12.2 ± 3.41	12.3 ± 3.2	N/S
HOMA – IR (µU/ml-mmol/l)	4.23 ± 1.7	4.31 ± 1.8	N/S

N/S for P value – not statistically significant

There was no statistically significance between two groups at baseline. HRT was associated with statistically significant decreases in serum fasting glucose, HbA1C, insulinemia levels and HOMA – IR values in the DM - HRT group. There was no significant reduction in glucose levels and HbA1C together with no significant increase in insulinemia levels and HOMA-IR values in the DM non-HRT group throughout 12 months.

Regarding group comparison after 12 months, there was statistically significance noted in all examined parameters. Namely, there was statistically significant increase of FPG, HbA1C, insulinemia and HOMA - IR between DM – HRT group and DM – non HRT group after 12 months. The changes in serum fasting glucose, HbA1c levels, fasting insulin levels and HOMA-IR are given in Table 2.

**Table 2:** Effects on HRT on Fasting Plasma Glucose (FPG), HbA1C, Insulinemia & HOMA – IR

	Women on HRT (*n* = 20)	P value	Women not on HRT (*n* = 20)	P value	P* value
FPG (mmol/l)				
Baseline	7.8 ± 0.86	p< 0.001	7.8 ± 1.1	P=0.66	P*< 0.0001
12 months	6.9 ± 0.6		8.0 ± 0.9		
HbA1C %				
Baseline	7.6 ± 0.54	p<0.001	7.9 ± 0.5	p=0.477	P*< 0.0001
12 months	7.2± 0.43		7,7 ± 0.4		
Insulinemia (µU/ml)					
Baseline	12.2 ± 3.41	p<0.001	12.3 ± 3.2	p= 0.08	P*<0.0001
12 months	10.4 ± 2.92		13.1 ± 3.7		
HOMA – IR (µU/ml-mmol/l)					
Baseline	4.23 ± 1.7	P<0.001	4.31 ± 1.8	P=0.69	P*<0.0001
12 months	3.18 ± 1.4		4.54 ± 1.7		

P < 0.05 statistically significant for all postmenopausal women included in adequate group at baseline and after 12 months;

* P < 0.05 statistically significant for group comparison at 12 months.

## Discussion

The results of the current study indicate that use of HRT in postmenopausal women contributes to the variability in insulin sensitivity observed in diabetic postmenopausal women. Specifically, women taking oral estrogen plus progesterone have higher glucose utilization and insulin sensitivity than women who were not on HRT. Similar to our findings, several studies suggest that estrogen replacement therapy improves glucose homeostasis [29-31]. HRT - treated women had significantly lower fasting insulin levels than women not taking HRT [32]. Glucose utilization by the hyperinsulinemiceuglycemic clamp had a tendency to increase, and HbA1c significantly decreased after 3 months of oral estradiol therapy in postmenopausal women with type 2 diabetes and moderate hyperandrogenicity [33]. The dose of therapy may be important such that low doses of conjugated equine estrogen improved insulin sensitivity by ITT, but higher doses resulted in deterioration of insulin sensitivity [34].

Other studies report that estrogen therapy does not affect carbohydrate metabolism [14-16, 35-37]. In the Postmenopausal Estrogen/Progestin Interventions Trial [14], women were given estrogen alone or one of three estrogen/progestin regimens for 3 years. Fasting insulin levels decreased nonsignificantly in women on active treatments, and changes in 2-h insulin did not differ between treatments or with the treatments. Furthermore, estrogen replacement therapy did not change HbA1c and insulin area under the OGTT curve in patients with diabetes [35, 36] or change glucose tolerance or glucose uptake by the hyperinsulinemiceuglycemic clamp in healthy postmenopausal women [15, 16]. Additional reports conclude that estrogen therapy may, in fact, worsen glucose homeostasis [32–35]. Randomized trials of postmenopausal women indicate that 6–18 months of HRT increase fasting insulin levels [33] and decrease insulin sensitivity using either the intravenous glucose tolerance test (IVGTT) [34] or the insulin tolerance test (ITT) [35].

Differences in the study population (healthy versus diabetic postmenopausal women), type of therapy (estradiol versus the combination of estradiol plus progestin), and method of measuring insulin sensitivity (fasting insulin concentrations, OGTT, ITT, IVGTT, glucose clamp) may explain the disparate results of HRT on glucose metabolism. Moreover, the route of estrogen administration may contribute to discrepancies in findings such that transdermal therapy may [15] or may not affect glucose metabolism [36, 37]. However, because none of the women in our study were using transdermal estradiol therapy, this does not contribute to the differences in glucose utilization observed.

The mechanism by which estrogen treatment may alter insulin action in humans is not completely understood. Similar to our results, animal studies would suggest that estradiol maintains or improves insulin sensitivity. Estrogen has been shown to increase glucose transport and glucose utilization in muscle cells of animals [38, 39]. Estrogen regulates insulin-induced glucose transport [40] through glucose transporter translocation in rat skeletal muscle [41]. Moreover, in oophorectomized rats, there is a reduction in insulin-stimulated translocation of GLUT4 to the plasma membrane as well as a reduction in glycogen synthase protein expression in skeletal muscle, which contributes to a decrease in whole-body insulin sensitivity [41]. We are unaware of any studies examining skeletal muscle glucose transport in postmenopausal women on or not on HRT. Based on the literature, which suggests that GLUT4 levels do not vary between normal lean glucose-tolerant and obese diabetic subjects [42], we would not expect differences in skeletal muscle GLUT4 protein between women taking estrogen, on HRT, or not on HRT. However, it is possible that glycogen synthase, GLUT4 translocation, and/or other early steps in the insulin-signaling pathway change are altered with the use of estrogen or estrogen plus progesterone in women. In addition, another potential mechanism of estrogen on insulin sensitivity could be mediated through estrogen’s effect on peripheral vascular reactivity [43, 44].

In conclusion, our results show that women taking oral HRT are more insulin sensitive than non-hormone users. Additional studies with longer duration and more subjects are needed to determine the cellular mechanisms that could account for the differences in insulin sensitivity between postmenopausal women who are taking estrogen, on combined hormonal therapy, or not on HRT.
